# Creating a medical English-Swedish dictionary using interactive word alignment

**DOI:** 10.1186/1472-6947-6-35

**Published:** 2006-10-12

**Authors:** Mikael Nyström, Magnus Merkel, Lars Ahrenberg, Pierre Zweigenbaum, Håkan Petersson, Hans Åhlfeldt

**Affiliations:** 1 Department of Biomedical Engineering, Linköpings universitet, SE-58185 Linköping, Sweden; 2 Department of Computer and Information Science, Linköpings universitet, SE-58183 Linköping, Sweden; 3 Assistance Publique-Hôpitaux de Paris, F-75683 Paris Cedex 14, France; 4 Inserm, U729, F-75270 Paris Cedex 06, France; 5 Inalco, CRIM, F-75343 PARIS Cedex 07, France

## Abstract

**Background:**

This paper reports on a parallel collection of rubrics from the medical terminology systems ICD-10, ICF, MeSH, NCSP and KSH97-P and its use for semi-automatic creation of an English-Swedish dictionary of medical terminology. The methods presented are relevant for many other West European language pairs than English-Swedish.

**Methods:**

The medical terminology systems were collected in electronic format in both English and Swedish and the rubrics were extracted in parallel language pairs. Initially, interactive word alignment was used to create training data from a sample. Then the training data were utilised in automatic word alignment in order to generate candidate term pairs. The last step was manual verification of the term pair candidates.

**Results:**

A dictionary of 31,000 verified entries has been created in less than three man weeks, thus with considerably less time and effort needed compared to a manual approach, and without compromising quality. As a side effect of our work we found 40 different translation problems in the terminology systems and these results indicate the power of the method for finding inconsistencies in terminology translations. We also report on some factors that may contribute to making the process of dictionary creation with similar tools even more expedient. Finally, the contribution is discussed in relation to other ongoing efforts in constructing medical lexicons for non-English languages.

**Conclusion:**

In three man weeks we were able to produce a medical English-Swedish dictionary consisting of 31,000 entries and also found hidden translation errors in the utilized medical terminology systems.

## Background

The development of health care information systems supporting manual or automatic data processing calls for the use of medical terminologies[1]. Coding into controlled vocabularies as well as free text indexing of e.g. patient records rely on lexical resources as they normally involve term matching. Medical lexicons are available in English [2], but in a globalized health care system, these resources also need to be internationally available and accepted. Work on medical lexicons are ongoing e.g. for German [3] and for French [4]. In Sweden it is mandatory to use Swedish versions of statistical medical classifications, for instance a Swedish version of the ICD-10, in reports to the National Board of Health and Welfare. Due to Swedish regulations all health records must also be written in Swedish. Consequently, Swedish versions of the international medical terminology systems are needed.

Until now translation of international medical terminology systems into Swedish has been done manually. Large resources have therefore been invested even in translations of smaller terminology systems. From personal communication with Lars Berg, head of the Unit for Classifications and Terminology at the Swedish National Board of Health and Welfare we were informed that the total cost of translating ICF, which contains 1,495 terms, is estimated to € 110,000 (1,030,000 SEK). This includes a first beta translation and the following revisions and validations by groups of health care professionals. If larger terminology systems, like SNOMED CT with its one million descriptions, are going to be translated manually, the cost will be much higher.

Consequently, from the perspective of a relatively small European country such as Sweden, there is a great interest in the possibility of reducing translation costs through the use of semi-automatic translation methods. However, to work effectively these methods need in general a bilingual dictionary (in this case an English-Swedish dictionary) suitable for the medical terminology domain[5]. The medical dictionaries that already exists are made for the general medical domain and not adapted for the stricter medical terminology systems domain. Some translation methods of interest, such as statistical methods, are possible to use if the dictionary also contains phrases with the words or co-existence information of the words[5]. Currently, there is no English-Swedish medical dictionary suitable for medical terminologies where also phrases or co-existence information in general is included, but there are several medical terminologies such as ICD-10, ICF and MeSH that have official translations from English to Swedish. These medical terminology systems can be used as a basis for building a medical dictionary adapted for medical terminology systems. The resulting medical dictionary would include terminology phrases where the dictionary's words are used.

### Objective

The objective of this paper is to report on the process of creating a medical English-Swedish dictionary using interactive word alignment. The first step of the project is composition of a terminology collection composed from five terminology systems available in both English and Swedish. The second step is creation of a medical English-Swedish dictionary using interactive word alignment of the terminology collection. The paper also describes the composed terminology collection and the ITools suite used in the word alignment process. Further, the resulting dictionary of term candidates is presented and analysed with respect to the effect of the interactive word alignment process. The third step, standardization of the dictionary's terms, and the fourth step, use of the dictionary as a resource for semi-automatic translations, are briefly discussed together with future inclusion of the term candidates into a multi-lingual medical dictionary. We also report on translation errors in the utilized medical terminology systems that were found during the study.

The methods presented in this paper are not only relevant for the language pair English-Swedish, but for most other West European language pairs like English-Spanish and English-French as well. The contribution is briefly discussed in relation to other ongoing efforts in constructing medical lexicons for non-English languages.

## Methods

### Terminology Collection

From electronic sources with varying format we have composed a collection of five medical terminology systems that we call Terminology Collection (TermColl). The hierarchical structure of the medical terminology systems has been kept in TermColl, and TermColl contains the terminology system's rubrics in both English and Swedish. By rubric we mean the short informative term accompanying each code in the terminology system. For this study we have extracted the rubrics that exist in parallel in English and Swedish. For some of the codes in the terminology systems, both main rubrics in English and Swedish and synonym rubrics in English and/or Swedish can be found. In these cases we have included the English and Swedish main rubrics and excluded all synonym rubrics from the study. An example is the English rubric 'Enteropathogenic Escherichia coli infection' and the Swedish rubric 'Infektion med tarmpatogena Escherichia coli-bakterier' accompanying the ICD-10 code A04.0. Table 1 gives the number of rubrics and the average length of the rubrics in TermColl's terminology systems. These systems are described below.

**Table 1:** TermColl's contents. Number of rubrics in parallel and average number of words per (standard deviation of) rubrics from the different terminology systems in TermColl.

Terminology	Rubrics	Average number of words per (standard deviation of) English rubric	Average number of words per (standard deviation of) Swedish rubric
ICD-10	11,503	4.9 (2.8)	5.3 (3.4)
ICF	1,495	4.2 (2.5)	4.2 (2.8)
MeSH	19,081	1.8 (0.8)	1.4 (0.7)
NCSP	5,523	6.7 (3.1)	5.7 (2.8)
KSH97-P	967	3.9 (2.5)	3.5 (2,4)

### Content of the Terminology Collection

#### ICD-10

International Statistical Classification of Diseases and Related Health Problems, Tenth Revision, ICD-10, is provided by WHO[6]. The classification is a statistical classification with the purpose of enabling systematic description and comparison of mortality and morbidity data between different areas and/or over time. The classification is in practice the international standard for general epidemiological purposes[7]. The Swedish National Board of Health and Welfare is responsible for the Swedish translation[8].

#### ICF

International Classification of Functioning, Disability and Health, ICF, is another terminology provided by WHO[9]. Its purpose is to be a framework for describing health and health-related states such as what a person with a given disease is able to do in different situations. The Swedish National Board of Health and Welfare is responsible for the Swedish translation[10].

#### MeSH

Medical Subject Headings, MeSH, is provided by United States National Library of Medicine and is a controlled vocabulary used for example in MEDLINE for indexing the content of biomedical papers, books and documents[11]. The version included in TermColl is the year 2003 version. The library at Karolinska Institutet is responsible for the Swedish translation[12].

#### NCSP

NOMESCO Classification of Surgical Procedures, NCSP, is a common Nordic classification of surgical procedures used for comparing the surgical activities in Denmark, Sweden, Finland, Norway and Iceland[13]. It was created on initiative from and first published in English by the Nordic Medico-Statistical Committee (NOMESCO) and is annually updated by the Nordic Centre for Classifications in Health Care. The version included in TermColl is the year 2004 revision 1 version. NCSP was published in Swedish in 1996. The Swedish National Board of Health and Welfare is responsible for the Swedish translation[14].

#### KSH97-P

Primary Health Care Version of The International Statistical Classification of Diseases and Related Health Problems, KSH97-P, is a statistical classification for the Swedish primary health care provided by the Swedish National Board of Health and Welfare, and it is derived from the Swedish version of ICD-10[15]. Parts of its rubrics are identical with rubrics in ICD-10, while other rubrics are aggregates for rubrics in ICD-10. The English translation is made available by the Swedish National Board of Health and Welfare[16].

### Characteristics of the Terminology Collection

The translations of the main rubrics are done with the intention that the associated codes will be used for the same purposes independent of the rubric's language. The rubrics are therefore often direct translations or close translations of each other. An example of a direct translation is 'Orientation to time' and 'Orientering till tid' (literally 'Orientation to time') accompanying the ICF code b1140. An example of a close translation is 'Personal history of other specified conditions' and 'Andra specificerade tillstånd i den egna sjukhistorien' (literally 'Other specified conditions in the own illness history') accompanying the ICD-10 code Z87.8.

In some cases the rubrics are freely translated, but mean the same thing. This is particularly the case when there are different traditions in the healthcare system to express something in English and Swedish. An example is 'Excision of segments II, III and IV of liver' and 'Vänstersidig hemihepatektomi' (literally 'Left side hemihepatectomy') accompanying the NCSP code JJB40.

In some cases information in the rubrics is implicit in one language and explicit in the other. An example is 'Heart' and 'Malign tumör i hjärtat' (literally 'Malignant neoplasm of heart') accompanying the ICD-10 code C38.0. Here the information 'Malignant neoplasm of' in the English rubric is implied from the parent rubric (C38) 'Malignant neoplasm of heart, mediastinum and pleura'. In the Swedish rubric is it stated explicitly. This explicit information was added by the Swedish National Board of Health and Welfare during the translation process.

Of the 39,500 rubric pairs included in this study 8,000 rubric pairs contain only one word in each language. Most of these rubric pairs have medical content (e.g. disease names, anatomical names, drug names and chemical names). Others refer to aspects of daily life and geographical names.

### Word alignment

Since the beginning of the nineties parallel texts, i.e. collections of texts with their translations, have been explored as sources of translation data at the word and sentence levels. While the corresponding rubrics of TermColl are generally not sentences, and do not combine to form texts, they are, for the most part, translational equivalents. They can therefore be explored in the same way to find correspondences among smaller segments, in particular among words and multiword units that can serve as lexical units for translation of medical terminology systems. As is common practice, we use the term word alignment both for referring to the process of discovering such correspondences, and for referring to a particular correspondence found between an English and a Swedish word-level unit.

Most word alignment systems use co-occurrence statistics as a basis for making decisions about correspondences. The underlying intuition is that pairs of units that are translations of each other are more likely to appear in corresponding regions of a parallel text than are other pairs. In addition, language-specific data, such as bilingual dictionaries, or assumptions about grammatical constraints on words that are translational equivalents can be used in the alignment process[17]. By combining data of different types in an optimal fashion, significant improvements can be made in comparison with using a single measure[18]. Especially for large parallel corpora, parameter estimation of statistical models, as used in the popular Giza++ system [19], has proved effective for word alignment.

### Alignment tools

**Figure 1:**
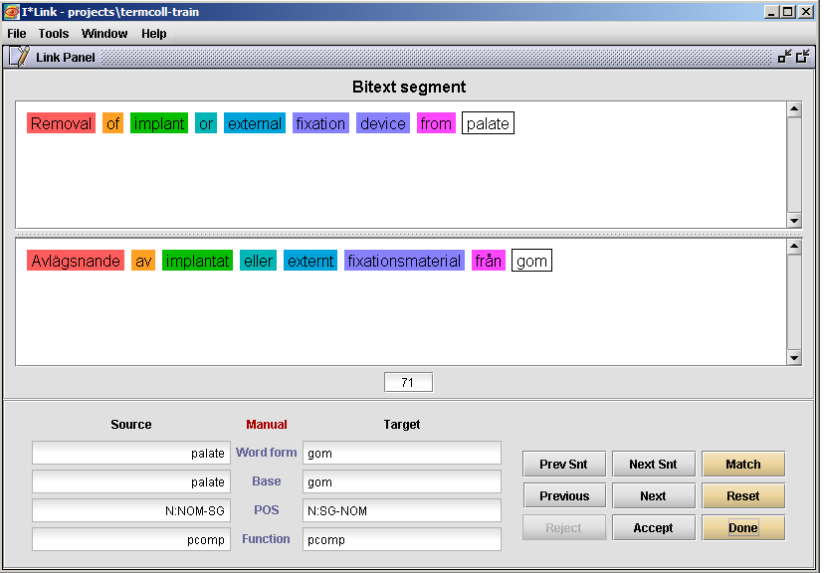
**ILink screen shot**. Screen shot of a portion of the interactive linker ILink – the Link panel window. Here a sentence pair in English and Swedish is active and an interactive alignment is taking place. So far seven alignments have been made (*Removal-Avlägsnande, of-av, implant-implantat, or-eller, external-externt, fixation device-fixationsmaterial, and from-från*). The current suggestion is *palate-gom *to which the annotater can respond with Accept or Reject (or go back and change other alignments). When the alignment of the sentence pair is complete, the button Done is pressed and all decisions made by the annotator are stored as training data on four different levels (word form, base form, parts-of-speech and function) which are indicated in the lower left corner of the screen shot. These dynamic resources are later used by the automatic aligner ITrix.

**Figure 2:**
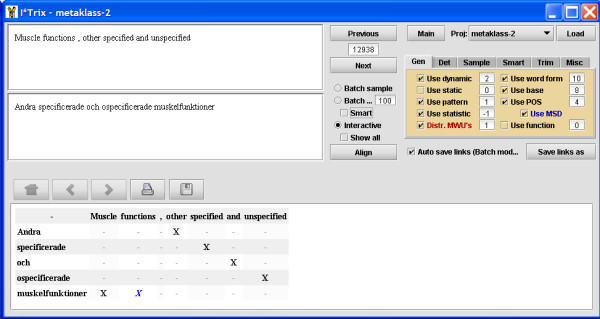
**ITrix screen shot**. Screen shot of the automatic aligner ITrix displaying results of word alignment in one sentence pair. The source and target sentences are shown in the top left corner and the word alignment results are given below in a matrix. Each detected word alignment is signalled by an 'X' in the matrix. Multiple word unit alignments are signalled by multiple X's on the same line, such as the English 'Muscle functions' which has been aligned with the Swedish single word 'muskelfunktioner'. In the multiple tab box on the right hand side, weights for how different resources should be combined can be configured.

**Figure 3:**
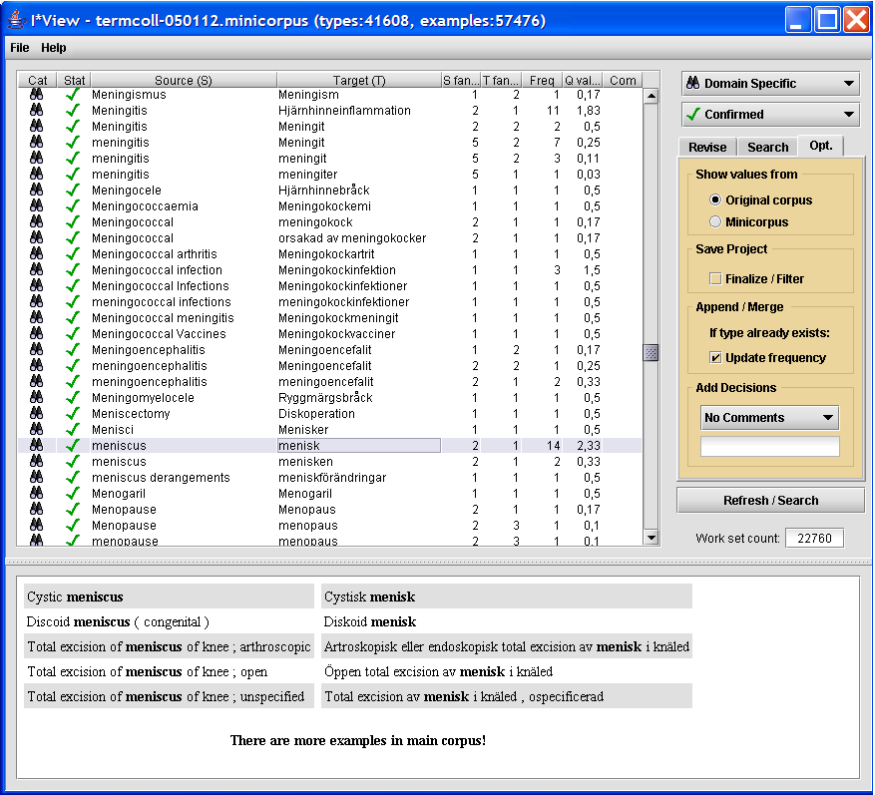
**IView screen shot**. Screen shot of the browser IView displaying confirmed domain-specific pairs. The main window displays the word alignments in a table format, giving the source word(s), target word(s), source fan out (number of different word pairs the source word appears in), target fan out (number of different word pairs the target word appears in), frequency of the alignment, and a quality value (Q val), which is used to rank the pairs during verification. The annotator uses IView to confirm correct alignments and to remove erroneous alignments on the type level.

The software used for this project is the ITools suite, which includes tools for interactive training (ILink), automatic alignment (ITrix), and a viewer for editing and browsing alignment data (IView) [20-23]. A screen shot from the interactive aligner ILink is shown in Figure 1, while the automatic aligner ITrix is shown in Figure 2 and the browser IView is depicted in Figure 3. The ITools suite also includes functions for sampling test and training data sets, automatic evaluation and statistical processing.

The basic approach used for alignment in the ITools suite combines evidence from various sources by assigning each piece of evidence a score and then calculating a joint score for all of them (cf. [18]). The user can influence the scoring by assigning different weights to different types of evidence. The evidence is based on static resources such as bilingual dictionaries and part of speech patterns across languages, which do not change by training, and dynamic domain- and application-specific data that is created through interactive training and machine learning. In addition, evidence such as string similarity (cognates) can be used.

The ITools suite is supported by Connexor's Machinese Syntax parsers [24] which provided the grammatical information for English and Swedish used in this project.

Our word alignment process consisted of the following steps:

1. Morphological, syntactic and dependency analysis resulting in XML markup

2. Calculation of co-occurrence statistics

3. Sampling test and training data sets

4. Creating a gold standard for testing

5. Training, i.e. creating dynamic resources interactively by reviewing alignment proposals

6. Running automatic alignment

7. Automatic evaluation using test data

8. Further training

9. Tuning and second run of automatic alignment

10. Evaluation

11. Verification and categorization

In step 1 the sentence-aligned source and target text were parsed independently using the Machinese Syntax parsers for English and Swedish. The tagged texts were then converted to an XML-format containing linguistic information not only on parts-of-speech categories, but also on morphosyntactic features, as well as syntactic functions (subject, object, adverbial, etc.) and syntactic dependencies (e.g. head words and modifiers).

In step 2 statistical resources were created both for the word form level (inflected words) and lemma level (base forms). The statistical resources were calculated with significant t-score and dice associations on co-occurrences between items in the source and target texts, yielding four different static dictionaries to be used in the interactive and automatic word alignment steps further on. In the third stage, test set of five hundred rubric pairs was randomly sampled from TermColl as well as a training set consisting of 4,200 rubric pairs.

In step 4 the test set was interactively aligned using the interactive aligner ILink, thereby creating a gold standard, to be used in the evaluation (step 7). In step 5, a training environment was set up in the interactive ILink tool where the training set was used as input, and where the training resulted in resources on four levels: 1) the word form level, 2) the base (lemma) level, 3) the parts-of-speech level, and 4) the syntactic function level. These resources are dynamic in the sense that they are augmented as interactive alignment proceeds.

ILink proposes word alignments step-by-step and then the annotator accepts or rejects the proposals until all possible alignments in a rubric pair have been completed. Then all decisions made for the active pair are stored in the dynamic resources and the process continues to the next sentence (see Figure 1). Every acceptance of an ILink alignment results in positive training data on all four levels mentioned above. A rejection of an alignment proposal will result in negative data. Both positive and negative training data are taken into account by the automatic alignment module in determining the final score, sentence by sentence. As the dynamic resources are filled with new data for each completed rubric pair, the speed of the process of interactively deciding the word alignments is continuously increasing. Not all of the training set was processed in step 5; instead only a third of the training set was used before we moved on to step 6 where the automatic aligner was run. In the sixth step the automatic aligner ITrix was applied on the test set utilising the resources built up during the preceding training (see Figure 2). By evaluating the test set against the gold standard, slight adjustments of how ITrix was configured were made, e.g. it is possible to set different weights to different resources, and turn on or off certain heuristics. Then it was possible to proceed to step 8 where the rest of the training set was interactively aligned with ILink, thereby increasing training data further.

In step 9 the final automatic alignment was performed using ITrix and then (step 10) the test set was evaluated against the gold standard. The evaluation of the final ITrix run was then compared to a baseline run (where only statistical data were used) and to one where no training data had been utilised (see Table 2 Alignment evaluation).

**Table 2:** Alignment evaluation. Evaluation data performed on the token level alignment of the parallel text.

Configuration	Recall	Precision	F-measure
Baseline (only statistics)	0.51	0.75	0.61
Statistics + static data + patterns - no training data	0.67	0.65	0.66
Including training data	0.77	0.76	0.76

However, to arrive at a usable term collection, the output from the word alignment needed to be verified and this was performed in step 11. IView was used for the last step (see Figure 3), which consists of verifying extracted term pairs with access to sample contexts as well as statistical data. In IView all the token alignments made by ITrix are compiled into a table of translation pair types in a graphical environment where the annotator can confirm translation pairs as terms or as belonging to 'general language', i.e., they are correct alignments but cannot be considered as terms in the medical domain. To make the process quicker, the annotator can sort the alignment data in different ways, e.g. by utilising a quality value (Q val) based on mutual information and rewards consistent translations with high frequency [22]. Alignments with low scores are typically pairs with low frequency where the source and target words occur in many other alignment pairs. Erroneous alignments are removed and the result is a set of accurate word alignments from TermColl. The parts of the process that are most time-consuming are the training sessions (step 4, 5 and 8) and the verification/categorization (step 11).

In principle, other word alignment systems, such as the already mentioned Giza++ [19] or the Uplug system [25], could be used for the same purpose and with similar results as the aligners used in this project. An advantage of the ITools suite is, though, that its different components share formats and data structures and communicate well with one another. Thus, the data created interactively in the training steps are smoothly transferred to the automatic system to boost its performance.

### Performance measures

The performance of the alignment process is evaluated by recall and precision scores based on a test set that was created by manual word alignment early in the project. Here the focus is on token alignments, i.e., the scores are given for the actual alignments made on the tokens (i.e., each word occurrence) in the text collection. Recall (R) is calculated as the number of proposed alignments (A) included in the reference alignments (S) in relation to the number of reference terms in the manually created gold standard. Precision (P) is then the percentage of correct alignments in relation to the proposed alignments.

R=A∩SSP=A∩SA
 MathType@MTEF@5@5@+=feaafiart1ev1aaatCvAUfKttLearuWrP9MDH5MBPbIqV92AaeXatLxBI9gBaebbnrfifHhDYfgasaacH8akY=wiFfYdH8Gipec8Eeeu0xXdbba9frFj0=OqFfea0dXdd9vqai=hGuQ8kuc9pgc9s8qqaq=dirpe0xb9q8qiLsFr0=vr0=vr0dc8meaabaqaciaacaGaaeqabaqabeGadaaakeaafaqabeqacaaabaacbmGae8NuaiLaeyypa0ZaaSaaaeaacqWFbbqqcqGHPiYXcqWFtbWuaeaacqWFtbWuaaaabaGae8huaaLaeyypa0ZaaSaaaeaacqWFbbqqcqGHPiYXcqWFtbWuaeaacqWFbbqqaaaaaaaa@3B11@

The F-measure is the harmonic mean of recall and precision. The figures given in the next section are based on these formulas, which only focus on perfect alignments (i.e. multiword alignments must have a perfect match to be considered). Partially correct alignments (where parts of a multiword expression are correctly aligned) are ignored. Another way of illustrating the performance of the process is to present the number of alignment types (dictionary-like entries) that have passed the final verification stage mentioned above, i.e., the size of the proposed dictionary.

### Related work

Baud et al. [26] also aimed at building a bilingual medical lexicon by aligning words in a parallel medical terminology: the English and French ICD-10. However, they did not have access to the word alignment tools available today, and instead used the following two methods. The first one bootstrapped the alignment on single-word terms, and then propagated these alignments to pairs of two-word terms where one word is already known (either from the previous step or from an external bilingual lexicon). Additional such iterations to longer term pairs produced more word alignments. The second method relies on the distributional profiles of words in terms, and pairs words that have the most similar distributional profiles. No syntactic parsing was performed; however, words were lemmatized, grammatical words ('stop words') were removed, and words were decomposed into subwords [27]. This allowed the authors to obtain nearly 10,000 alignments, with an estimated 98 % precision. However, no recall figures are reported, which makes comparison unachievable.

Instead of exploiting medical terminologies, Déjean et al. [28] used both parallel and comparable corpora of article abstracts to extend the German MeSH and applied the resulting bilingual lexicon to a cross-language information retrieval task. They do not report, however, using the MeSH itself as a parallel resource for alignment of shorter terms.

Deléger et al. [22] applied the same word alignment methods as the present work to a corpus of parallel English-French medical texts obtained from the Web. This provided larger volumes of input than the available parallel medical terminologies. The result was 91,000 alignments, but only 10,000 were considered 'medical'. (The criterion was that the English term in the alignment must be present in SNOMED CT or MeSH, two large medical terminologies.)

As mentioned in the introduction, there are monolingual medical lexicons for English [2], German [3] and French [4] and a method to create multilingual lexicons consists in mapping monolingual lexicons to one another. This is the goal of ongoing work in SemanticMining [29], where each term in each lexicon is mapped to an 'interlingua' representation using a morpho-semantic term normalization engine. This kind of method, which relies on internal term content, is complementary to that presented here, which leverages external knowledge of terms.

## Results

### Word alignment

All the eleven standard steps for carrying out alignment described in the section on ITools were used. We created a test set consisting of 500 rubric pairs (containing 1,615 token alignments) randomly sampled from the entire text collection. The test set was then used as a gold standard in the evaluation of the output of the automatic alignment.

Table 2 presents evaluation figures on recall, precision, and F-measure on three different configurations of the alignment. The first alignment was a baseline version where only statistical data were used as input resources. This version was made in order to emulate a purely statistical system. In the second run, made in order to illustrate performance before the actual training took place, all static resources such as general bilingual dictionaries, standard part of speech correspondence patterns, as well as statistical data, were utilised. However, no training data were used in this second run.

The training was made using ILink by one domain expert on 4,200 rubric pairs from TermColl excluding the MeSH portion. The mentioned domain expert who did the training was not the same annotator who created the gold standard. The quality of the translation correspondences in MeSH was generally so high that we did not need to use it for training the ITools suite; the automatic alignment performed well without training. The third and final run used training data from the interactive sessions and these results are also shown in Table 2. As can be seen the training sessions did substantially increase the performance.

By using the IView tool we processed the automatically generated term pair candidates in a categorization stage. This resulted in 31,000 confirmed pairs where 27,000 pairs were domain-specific and 4,000 were categorized as belonging to general language. Table 3 shows the number of confirmed term pairs after the categorization stage divided into groups by the number of words in the terms.

**Table 3:** Confirmed term pairs. Number of confirmed term pairs after the categorization stage divided into groups by the number of words in the terms.

		English	
		
		One word	Multiple words
	One word	19,396	9,738
Swedish			
	Multiple words	905	958

The categorization stage also resulted in 5,000 rejected pairs because the automatic alignment was only partially correct and 2,000 rejected pairs because the automatic alignment was completely wrong.

### Found translation errors

During the categorization step in IView we found some cases that seemed to be correctly aligned according to how the rest of the rubrics were aligned, but the aligned words themselves were not equivalent in the two languages. Evaluation showed that the errors stemmed from the original translation of the terminologies and not the word alignment. Nearly all of these rubrics were collected from NCSP. All errors originated from around 40 different problems in the translation, but some of them were included in more than one rubric, making the total number of errors higher. One example of a problem was that the English prefix 'allo' was translated into the Swedish prefix 'homo' in the rubrics dealing with transplantations. Another problem was that the English word 'partial' was sometimes absent in the Swedish translation. These errors have been documented for further analysis and corrections of the translations.

### Used human resources

In total we spent less than three man weeks on training and final categorization. The training took one week and the categorization work done in IView took less than two weeks.

## Discussion

### Multi-lingual medical dictionary generation

The current work is performed within the framework of a large-scale European research network entitled Semantic Interoperability and Data Mining in Biomedicine, SemanticMining, with one subgoal of developing a multi-lingual medical dictionary together with an interchange format for lexical information. This work is described in [30]. The generated term pairs are also going to be included in this dictionary.

The moderate training time of around one man week increased the performance and quality of the word alignment substantially compared to when a purely statistical system or the ITools suite without training were used. Even small increases in recall on the token level result in substantial increases in the number of generated term pairs for the dictionary. This means that when the quality of the token alignment is increased the quality of the dictionary is increased even more.

Spending less than two weeks on verification of the generated term pair candidates resulted in 31,000 confirmed term pairs. Obtaining the same result in this amount of time using a manual approach would have been impossible. With a purely statistical system term candidates could have been generated faster, but validation would have been much slower and no linguistic information associated with terms would have been captured. It is important to remember that the described process not only results in term translations but that it also entails the process of identifying possible term candidates as well as weeding out mistaken alignments and alignments which cannot be deemed as terms of the domain in question.

The fact that the main part (19,000 of 31,000) of the confirmed term pairs contains one word in both Swedish and English is reasonable because the intention of the word alignment is to create a dictionary. It is also reasonable that quite a large part (10,000 of 31,000) of the term pairs contains one word in Swedish and more than one word in English, because Swedish compounds are written as single words whereas English compounds are generally not.

### Relation to previous work

In the context of building multilingual medical lexicons, the present work is to our knowledge the first to apply advanced word alignment techniques to parallel medical terminologies. Previous work differs in the methods or material used to build bilingual lexicons. The two methods reported by Baud et al. [26] can be seen as emulating the basic functions of the tools used here. The methods used by Déjean et al. [28] need both parallel and comparable corpora for the processing.

Deléger et al. [22] use of the same methods as in the present work involved a larger input corpus and resulted in a larger volume of alignments. However, the density of medical words, even in medical Web sites, cannot be expected to be as high as that in medical terminologies. To find 10,000 aligned medical words of 91,000 aligned words in total is a much lower ratio than in the present work.

Extracting words from a corpus of texts requires a careful selection of these texts. Depending on this selection, different domains, genres and levels of language, to cite but a few, can be obtained and will influence the quality and relevance of the obtained word pairs. In contrast, focusing on terms in an official terminology directly selects professional language. This contrast of terminologies with corpora raises the question of word attestation. On the one hand, terminologies generally encode normative terms, and may have a slow evolution and update, whereas corpora composed of recent texts display potentially more up-to-date, actually used vocabulary. Depending on their composition, corpora may also be a source of 'lay vocabulary' (also called 'patient vocabulary', or 'consumer vocabulary') which is more difficult to find in present terminologies (a notable exception being the MedlinePlus Health Topics). On the other hand, terminologies are generally subject to careful review and maintenance, so that they contain very few errors or misspellings, whereas text corpora may display more errors. Finally, the choice of sources depends on one's objectives, and a selected combination of both kinds of sources may be the most appropriate for a given objective.

### Terminology inconsistency

A side effect of systematically revising the terminology systems in the fashion outlined in this paper is that translation errors could be discovered. NCSP was first published in Swedish in 1996 and has undergone annual revisions. Despite this, 40 different problems were found without designing the study to specifically search for inconsistencies. These results indicate the power of word alignment for finding inconsistencies in terminology translations.

A focus of interest in the future is an extended analysis of the translation inconsistencies, for example of when one word in the source language is translated into different words in the target language. Examples of this kind of inconsistencies are that the English word 'operation' has been translated into six different words in Swedish and that nine different English words have been translated into the Swedish word 'operation'.

The inconsistencies can be allowed variations, unnecessary variations, and incorrect variations. Allowed variations imply that the target words are accepted synonyms of each other or the source words have more than one meaning and this case requires no further explorations. Unnecessary variations imply that the target words are synonyms but that one of the target words would be considered as a preferred term. The knowledge of unnecessary variations can be used both in the dictionary standardization process described below and to improve the translations of the terminology systems used in TermColl. Incorrect variations imply that at least one of the target words is an incorrect translation and requires correction. Examples of incorrect variations are the above described errors in NCSP.

### Future dictionary standardization

A future step is to standardize the generated term pairs in the dictionary to be suitable for use in semi-automatic translation methods for medical terminology systems. To keep the nature of the original terminology systems in the translated versions it would be a benefit if one word in the source language was consistently translated into the same word in the target language, as far as it is possible. The main goal of the standardization process is therefore to reduce the number of corresponding synonyms among the term pairs to the most recognized alternative or alternatives. The project will then continue with the semi-automatic translation step using the created standardized dictionary.

### The dictionary as an approximation of the medical language

Coding of patient records into controlled terminologies offers a big challenge into which the medical dictionary may help to gain insight. Since it is constructed from a set of terminologies commonly used in the health care system, it can be seen as an approximation of the medical language as expressed by terminology systems used for abstraction and statistical classification. The result of a follow-up study of a large corpus of clinical records and the current dictionary would yield important understanding of the overlap or missing areas between clinical language used in patient records and the terminology collection.

However, terminologies do not necessarily reflect the clinical language used in medical records. The difference between source (record) and target (terminology) vocabulary may for example be categorized as spelling variants, synonymy or aggregation, and in order to understand the coding process, the various degrees of semantic distance between records and terminologies must be taken into account. A previous study of this kind can be found in [31], where relationships between terms in primary health care records were analysed in relation to the rubrics of assigned KSH97-P codes. That study indicated the importance of lexical tools as one basis for the coding process, and the split terms provided by the newly created dictionary can be put into continued research in that area.

### Future improvements

The results of the alignment process could possibly have been improved if certain sources of errors could be eliminated. First, the taggers used are tuned for analyzing full sentences, but most of the input material consists of sentence fragments or noun phrases which gives the tagging a higher error rate than usual. For English there is a version of the utilized tagger that takes noun phrases as the default category that could have given better tagging analyses. However, this kind of tagger is currently only available for English.

Secondly, the test data set and the training data set were created by different people, which may have resulted in that slightly different alignment strategies were used.

A third cause of errors could be that the input data (TermColl) should have been filtered in another way in order to detect rubrics that were non-standard or that contained poor translation correspondences. While the MeSH part gave good alignment without training, some subparts of TermColl gave translation correspondences with a low quality so they rather increased the error rate than the number of term candidates. Two causes of bad quality were the terminology inconsistencies described above and very free translations with substantial additions as well as exclusions.

The term candidates generated in this project are all in word form formats. Using the markup from the syntactic tagging we could have used the base (lemma) form instead and thereby reduce the number of candidate pairs. However, since the intended usage of the dictionary is translation we believe that the word form version will be more useful in the future. This remains to be tested.

## Conclusion

By spending three man weeks on alignment training and final categorization, we were able to produce a medical English-Swedish dictionary consisting of 31,000 entries. With the used alignment and categorization methods it was also possible to find hidden translation errors.

## Abbreviations

ICD-10 : International Statistical Classification of Diseases and Related Health Problems, Tenth Revision

ICF : International Classification of Functioning, Disability and Health

KSH97-P : Primary Health Care Version of The International Statistical Classification of Diseases and Related Health Problems (In Swedish: Klassifikation av sjukdomar och hälsoproblem 1997 – Primärvård)

MeSH : Medical Subject Headings

NCSP :  NOMESCO Classification of Surgical Procedures

NLM :  United States National Library of Medicine

NOMESCO : Nordic Medico-Statistical Committee

TermColl : Terminology Collection (The authors' collection of ICD-10, ICF, MeSH, NCSP and KSH97-P in English and Swedish.)

WHO : The World Health Organization

## Competing interests

Authors Magnus Merkel and Lars Ahrenberg are co-owners of the company Fodina Language Technology AB, which holds the rights to commercial exploitation of the ITools suite.

## Authors' contributions

MN has been responsible for acquisition of data (TermColl), has done all manual training in ILink and evaluated all term pair candidates in IView and has made contribution to the experiments with the ITools suite and the analysis. He has written the sections Background, Terminology Collection and Terminology translation errors, major parts of Abstract and Discussion and been the editor of the paper.

MM is responsible for the setup and application the ITools suite and the experiments, has written the section Performance measures, co-written the sections Alignment tools and Discussion and major parts of the Results section.

LA has contributed to the interpretation and presentation of the results, has written the section Word alignment and contributed writing to several other sections.

PZ has contributed to the methods and discussion regarding related work in constructing medical lexicons for non-English languages and the use of parallel corpora techniques.

HP and HÅ have substantially contributed in the design of the study, and in drafting and revising the manuscript.

All authors read and approved the final manuscript.

## Pre-publication history

The pre-publication history for this paper can be accessed here:


